# Effect of antimicrobial oral rinse use on prognostic scores in critically ill patients

**DOI:** 10.1590/1807-3107bor-2026.vol40.029

**Published:** 2026-06-01

**Authors:** Jessie Capobiango Soares de MOURA, Teresa Cristina Valente da ROCHA, Renata de Moura Cruz QUINTANILHA, Silvia Paula de OLIVEIRA, Diamantino Ribeiro SALGADO, Michelle AGOSTINI, Sandra Regina TORRES

**Affiliations:** (a) Universidade Federal do Rio de Janeiro – UFRJ, Clementino Fraga Filho University Hospital, Dental Clinic Service, Rio de Janeiro, RJ, Brazil.; (b) Universidade Federal do Rio de Janeiro – UFRJ, Clementino Fraga Filho University Hospital, Department of Internal Medicine, Rio de Janeiro, RJ, Brazil.; (c) Universidade Federal do Rio de Janeiro – UFRJ, School of Dentistry, Department of Oral Pathology and Diagnosis, Rio de Janeiro, RJ, Brazil.

**Keywords:** Intensive Care Units, Chlorhexidine, Oral Hygiene Index, Simplified Acute Physiology Score, Organ Dysfunction Scores

## Abstract

This study compared oral hygiene and prognostic scores in intensive care unit patients who were either exposed or not exposed to an antimicrobial oral rinse. Both the Simplified Acute Physiology Score (SAPS 3), and Sequential Organ Failure Assessment (SOFA) score - recorded on the day of admission (D1) and on the day of the oral examination (Dv) - were retrieved from electronic medical records. Oral data obtained through intraoral examination were used to calculate the Critical Patient Oral Hygiene Index (CPOHI). Eighty-four patients were included in the group exposed to the oral rinse containing chlorhexidine, cetylpyridinium chloride, and propolis extract (CCPG), whereas 42 patients were assigned to the reference group (RefG). Gingival inflammation (47.6% vs. 16.7%; p < 0.001) and spontaneous bleeding (9.5% vs. 0%; p = 0.004) were more frequent in the RefG than in the CCPG, respectively. Oral hygiene was satisfactory in 53.2% of patients, with no difference between groups. Median SAPS3 and SOFA Dv were worse in the RefG. Patients in CCPG with poor oral hygiene (6.0 (0-14)) showed worse SOFA Dv scores than those with good oral hygiene (1.0 (0-13), p=0.001). Intubation was the only variable negatively associated with all prognostic scores in the multivariate analysis. Critically ill patients exposed to the antimicrobial oral rinse showed better prognostic scores and a lower frequency of gingival inflammation and spontaneous bleeding, supporting the indication of the antimicrobial rinse in oral hygiene protocols for critically ill patients.

## Introduction

After admission to an intensive care unit (ICU), patients’ oral health often deteriorates,^1^ largely because of challenges in performing adequate oral care. The presence of an orotracheal tube, prolonged mouth opening, and medication-induced hyposalivation contribute to increased dental biofilm accumulation and tongue coating, leading to alterations in the oral microbiota and proliferation of respiratory pathogens.^2-4^ Thus, biofilm control is essential for the prevention of dental caries and calculus, periodontal complications, as well as oral soft tissue changes such as coated tongue and candidiasis.^5^ Most published studies on the oral hygiene of ICU patients report an association between chlorhexidine use and lower incidence of ventilator-associated pneumonia, one of the most common respiratory complications in ICU patients.^6-8^ To the best of our knowledge, no published studies have examined whether oral hygiene is associated with prognostic scoring systems used for ICU patients. These scores have been used to estimate disease severity, predict mortality, and assist in therapeutic and ethical decisions.^9,10^ Among the most frequently used ICU scoring systems are the Sequential Organ Failure Assessment (SOFA) score and the Simplified Acute Physiology Score (SAPS 3).^11,12^

A potential relationship may exist between oral hygiene and the deterioration of systemic conditions in ICU patients, highlighting the importance of strict oral hygiene in this population.^13-15^ The use of a 0.12% chlorhexidine-based antimicrobial rinse is expected to reduce dental biofilm accumulation and improve oral hygiene in these patients.^16,17^ The Critical Patient Oral Hygiene Index (CPOHI) may be used to assess oral health status and quality of oral hygiene during routine evaluations of critically ill patients.^18,19^

In this study, we hypothesized that enhancing oral hygiene with an antimicrobial rinse could improve prognostic scores. Therefore, the aims of the study were to evaluate and compare oral hygiene indices and prognostic scores between ICU patients exposed and not exposed to an antimicrobial oral rinse containing 0.12% chlorhexidine, cetylpyridinium chloride, and propolis extract (CCP) as part of the oral hygiene protocol.

## Methods

This was a case-control study of patients admitted to the general ICU of a university hospital in the city of Rio de Janeiro, Brazil, during the study period. Patients aged ≥ 18 years who had been hospitalized in the ICU for at least 5 days were included. Patients who could not be examined because of discomfort or physical or technical limitations were excluded. Data were collected from two groups of patients: a reference group (RefG) and a group exposed to a CCP rinse (CCPG) as part of the oral hygiene protocol. The RefG was evaluated during a period when the hospital’s oral hygiene protocol did not include the use of a CCP oral rinse in the routine oral care of ICU patients (October-November 2021). Patients in the CCPG were evaluated after a 30-day period following the acquisition and incorporation of the antimicrobial rinse into the hospital’s oral hygiene protocol (January–April 2022). The product used was Noplak Max® (Laboratório Daudt Oliveira Ltda, Rio de Janeiro, Brazil), an alcohol-free antimicrobial oral solution containing CCP.

**Figure 1:**
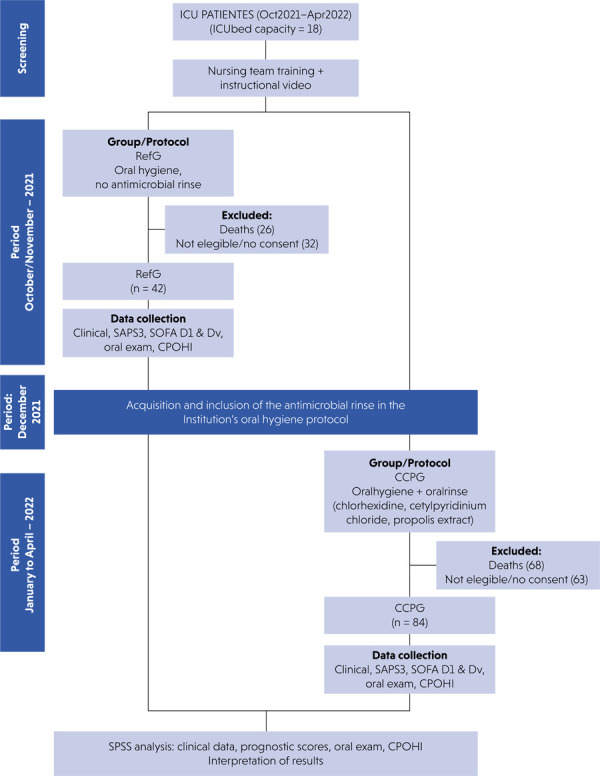
Study flowchart.

The study was conducted from October 2021 to April 2022, and 315 patients were hospitalized in the ICU during the data collection period. Ninety-four patients died and 95 did not meet the inclusion criteria or did not provide informed consent. Thus, a convenience sample of 126 patients was evaluated. The study flowchart is shown in Figure 1. The study was approved by the Research Ethics Committee of HUCFF/UFRJ (process number 47848621.3.00005257). The study was conducted in full compliance with ethical principles, including the Declaration of Helsinki (version 2002) and additional requirements set forth by the World Medical Association. All included patients or their guardians signed an informed consent form.

Demographic and clinical data (comorbidities, complications during hospitalization, medications, level of consciousness, intubation, type of nutrition, and length of ICU stay), as well as data required to calculate the SAPS 3 and SOFA prognostic scores, were collected from medical records and recorded in the study protocol form.

The SOFA score was calculated at two time points: within the first 24 hours of hospitalization (D1) and on the day of the intraoral examination (Dv). The SOFA score ranges from 0 to 24, with each organ system scoring from 0 to 4, according to the degree of organ dysfunction or failure. Higher scores indicate a worse prognosis. Variables such as oxygenation index, platelet count, use of vasoactive drugs, bilirubin level, Glasgow coma scale, and creatinine level were measured. The SOFA score may be calculated daily as needed.^11^

SAPS 3 is calculated using the following variables: 1) demographic variables (patient age); 2) comorbidities and reasons for ICU admission, which represent disease severity and the patient’s health status prior to hospital admission (i.e., pre-morbid condition); and 3) physiological variables, including the Glasgow coma scale, systolic blood pressure, heart rate, axillary temperature, oxygenation, arterial pH, total bilirubin, creatinine, leukocytes, and platelets). SAPS 3 scores range from 16 to 217 points, with higher scores indicating a worse prognosis. SAPS 3uses clinical and laboratory parameters measured within the first 24 hours of hospitalization to estimate ICU mortality.^10,1 2^

All patients underwent a bedside intraoral physical examination performed simultaneously by two dentists. Data obtained during the oral examination were recorded in the study protocol form. Wooden spatulas, sterile gauze, and a light-emitting diode (LED) flashlight (Vextron, São Paulo, Brazil) were used to perform the examinations. The collected data encompassed findings from the physical examination of oral soft tissues and teeth, salivary characteristics, and oral hygiene quality.

Oral alterations that may be influenced by poor oral hygiene include dental biofilm and calculus accumulation, coated tongue, candidiasis, gingival inflammation, and gingival bleeding.^20,21^ Some diagnoses were standardized, as follows. Coated tongue was defined as the presence of yellowish-white material on the dorsum of the tongue that could be removed with saline-soaked gauze.^22^ Dental biofilm was defined as the presence of a sticky, soft film covering the tooth surface.^23^ Gingival inflammation was recorded when the gingival mucosa was erythematous and edematous, with spontaneous bleeding or bleeding on probing.^24^ Salivary alteration was diagnosed when viscous, stringy, or sticky saliva, or mucosal dryness, was observed.^25^

The CPOHI classifies oral hygiene as satisfactory, deficient, or precarious. This index verifies the presence of biofilm, gingivitis, coated tongue, halitosis, secretion, blood, and food debris. Each item is assigned a value of 1 and their sum will determine the quality of oral hygiene: scores 0 to 1 indicate satisfactory hygiene; 2 to 3 denote deficient hygiene, and 4 to 7 correspond to precarious hygiene. For the purpose of analysis, oral hygiene was categorized into satisfactory (0 to 1) and unsatisfactory (2 to 7). The proposed index also accounts for the presence or absence of retentive factors, including orotracheal tube, Guedel cannula, orthodontic appliance, extensive caries, dental calculus, and dental prosthesis, which do not affect the score but reflect a greater difficulty in performing and maintaining proper oral hygiene.^18^ Professionals were properly trained and calibrated in the use of the CPOHI, achieving excellent inter-rater reliability (kappa: 0.824).^26^

To ensure standardization and proper execution of the oral hygiene protocols in ICU inpatients, the nursing team underwent training sessions conducted by the researchers, supplemented with an instructional video to support consistent application of the procedure (https://youtu.be/IVRvtJwidTM). The nursing staff performed oral hygiene every 12 hours, which included cleansing the oral structures (teeth, tongue, palate, vestibular fornix, floor of the mouth, and buccal mucosa) with gauze moistened with either distilled water (RefG) or the CCP antimicrobial solution (CCPG), with subsequent suctioning of fluids and lip moisturization.

The SPSS program (Statistical Package for the Social Sciences) version 13.0 (IBM Company Headquarters, New York, USA) was used for data storage and analysis. The distribution and frequency of the collected data are presented descriptively. Measurable variables were analyzed using the Mann-Whitney test, while categorical variables were compared between groups using the chi-square test. A general linear model (GLM) was used to evaluate the influence of independent variables on prognostic scores. To minimize the risk of type I error, significant levels were set at 1%.

## Results

The median age of patients was 66 years, ranging from 24 to 97 years. In the RefG, the median age was 66.5 (24–86) years, compared with 66.0 (24–97) in the CCPG. More than half of the patients were male (n = 74, 58.7%), comprising 69.0% (n = 29) in the RefG and 54.0% (n = 45) in the CCPG. Eighty-two patients (65.1%) were White, comprising 29 (69%) in the RefG and 53 (63.1%) in the CCPG. The remainder of the patients were Black. No statistically significant differences were found between the two groups in terms of age, ethnicity, and sex.

The most prevalent comorbidities were heart diseases (80.2%), followed by malignant neoplasms (32.5%) and diabetes mellitus (31.7%), with no significant differences in their frequencies between the groups. The most common complications were anemia (84.9%), cardiac complications (34.9%), and respiratory complications (28.6%), with no significant differences observed between the groups. The most frequently used medications during hospitalization were anti-ulcer drugs (94.4%), analgesics (81.0%), and insulin (81.0%). The clinical data for both groups are detailed in Table 1.

**Table 1:** Clinical characteristics of the 126 patients included in the study, according to study group, as retrieved from medical records.

Variables	Total		Reference group		CCPG		p-value
	n	%	n = 42	%	n = 84	%	
Comorbidities							
Heart diseases	101	80.2	33	78.6	68	81.0	0.752
Malignant neoplasms	41	32.5	18	40.9	23	27.4	0.080
Diabetes mellitus	40	31.7	12	28.6	28	33.3	0.588
Kidney diseases	22	17.5	7	16.7	15	17.9	0.868
Respiratory diseases	21	16.7	10	23.8	11	13.1	0.128
Neurological disorders	20	15.9	9	21.4	11	13.1	0.228
Gastric diseases	15	11.9	2	4.8	13	15.5	0.080
Liver diseases	14	11.1	3	7.1	11	13.1	0.316
History of COVID-19	12	9.5	1	2.4	11	13.1	0.053
Endocrine disorders	9	7.1	3	7.1	6	7.1	1.000
Hematological diseases	9	7.1	2	4.8	7	8.3	0.463
Genitourinary diseases	9	7.1	2	4.8	7	8.3	0.463
Autoimmune diseases	6	4.8	3	7.1	3	3.6	0.375
Allergic reactions	1	0.8	0	0	1	1.2	0.478
Complications during hospitalization							
Anemia	107	84.9	34	81.0	73	86.9	0.379
Cardiac	44	34.9	19	45.2	25	29.8	0.086
Respiratory	36	28.6	18	42.9	18	21.4	0.012
Renal	23	18.4	12	28.6	11	13.3	0.037
Sepsis	20	15.9	7	16.7	13	15.5	0.863
Gastrointestinal	16	12.7	5	11.9	11	13.1	0.850
Malignant neoplasms	9	7.1	5	11.9	4	4.8	0.142
Hematological	9	7.1	1	2.4	8	9.5	0.142
Neurological	6	4.7	4	3.1	2	1.5	0.076
Hepatic	5	3.9	1	0.8	4	3.1	0.519
Autoimmune	1	0.8	1	2.4	0	0	0.156
Endocrine	1	0.8	1	2.4	0	0	0.156
Diabetes mellitus	3	2.4	2	4.8	1	1.2	0.215
Medications							
Antiulcer drugs	119	94.4	40	95.2	79	94.0	0.783
Analgesics	102	81.0	30	71.4	72	85.7	0.969
Insulin	102	81.0	33	78.6	69	82.1	0.630
Anticoagulants	86	68.3	28	66.7	58	69.0	0.787
Antiemetic drugs	76	60.3	31	73.8	45	53.6	0.029
Psychopharmaceutical drugs	60	47.6	20	47.6	40	47.6	1.000
Antibiotics	50	39.7	21	50.0	29	34.5	0.094
Antihypertensive drugs	46	36.5	15	35.7	31	36.9	0.896
Corticosteroids	42	33.3	17	40.5	25	29.8	0.229
Vitamins	40	31.7	18	42.9	22	26.2	0.143
Antiarrhythmic drugs	38	30.2	17	40.5	21	25.0	0.074
Diuretics	32	25.4	14	33.3	18	21.4	0.148
Platelet antiaggregants	26	20.6	10	23.8	16	19.0	0.534
Neuroleptic drugs	20	15.9	9	21.4	11	13.1	0.228
Beta-blockers	7	5.6	5	11.9	2	2.4	0.028
Antifungal agents	7	5.6	3	7.1	4	4.8	0.582
NSAIDs	3	2.4	0	0	3	3.6	0.215
Antiviral drugs	3	2.4	1	2.4	2	2.4	1.000
Hypoglycemic drugs	2	1.6	1	2.4	1	1.2	0.614
Immunoglobulins	1	0.8	0	0	1	1.2	0.478

CCPG: chlorhexidine, cetylpyridinium chloride, and propolis extract group; NSAIDs: non-steroidal anti-inflammatory drugs; NS: Not significant.

Most patients were received oral nutrition, with 60.7% of them in the CCPG. Table 2 shows the level of consciousness, breathing, and nutrition for patients in both groups.

**Table 2:** Clinical characteristics of the 126 patients collected on the day of oral examination, according to study group.

Variable	Total		Reference group		CCPG		p-value
	n	%	n = 2	%	n = 84	%	
Level of consciousness							0.224
Alert	84	66.7	24	57.1	60	71.4	
Sedated, unresponsive	21	16.6	8	19.0	13	15.5	
Sedated, responsive	15	11.9	6	14.3	9	10.7	
Coma	6	4.8	4	9.5	2	2.4	
Oxygen delivery							0.022
Spontaneous	71	55.6	15	35.7	55	65.5	
IMV (orotracheal)	27	21.4	12	28.6	16	19.0	
O2 support	13	10.3	7	16.7	6	7.1	
IMV (tracheostomy)	12	9.5	7	16.7	5	6.0	
NIMV	3	2.4	1	2.4	2	2.4	
Nutrition							0.002
Oral	61	48.4	10	23.8	51	60.7	
Parenteral	40	31.7	20	47.6	20	23.8	
Enteral (nasogastric)	23	18.3	12	28.6	11	13.1	
G-tube	1	0.8	0	0	1	1.2	

CCPG: chlorhexidine, cetylpyridinium chloride, and propolis extract group; IMV: Invasive mechanical ventilation; NIMV: Non-invasive mechanical ventilation; G-tube: Gastrostomy tube.

No significant differences were observed between the groups regarding the percentage of patients who were intubated (28.6% in the RefG and 15.5% in the CCPG, p = 0.099) or tracheostomized (16.7% in the RefG and 6.0% in the CCPG, p = 0.103) at admission. The median length of stay in the ICU did not differ significantly between the groups: 6 days (range of 5 to 43 days) in the RefG and 7 days (range of 5 to 35 days, p = 0.992) in the CCPG.

The most prevalent oral mucosal findings were coated tongue (59.5%) and gingival inflammation (27.0%). Moreover, gingival inflammation (47.6% vs. 16.7%; p < 0.001) and spontaneous bleeding (9.5% vs. 0%; p = 0.004) were significantly more frequent in the RefG than in the CCPG. Viscous saliva (65.8%) and mucosal dryness (63.4%) were the predominant salivary alterations, with no statistically significant difference between the groups. Oral findings are shown in Table 3.

**Table 3:** Oral findings of the 126 patients, according to study group.

Variable	Total		Reference group		CCPG		p-value
	n	%	n = 42	%	n = 84	%	
Hygiene-related mucosal alterations*							
Coated tongue	75	59.5	26	61.9	49	58.3	0.700
Gingival inflammation	34	27.0	20	47.6	14	16.7	0.000
Spontaneous bleeding	4	3.2	4	9.5	0	0	0.004
Salivary alterations*							
Viscous saliva	83	65.8	28	66.7	55	65.5	0.894
Mucosal dryness	80	63.4	31	73.8	49	58.3	0.089
Dental alterations*							
Residual root	27	21.4	11	26.2	16	19.0	0.357
Caries	24	19.0	10	23.8	14	16.7	0.336
Fractured tooth	18	14.3	6	14.3	12	14.3	1.000
Tooth mobility	14	11.1	6	14.3	8	9.5	0.423
Dentition status							
Partially edentulous	85	67.5	28	66.7	57	67.9	0.446
Fully dentate	28	22.2	10	23.8	18	21.4	0.914
Completely edentulous	12	9.5	3	7.1	9	10.7	0.484
Hygiene (CPOHI)							0.456
Satisfactory	69	54.8	21	50.0	48	57.1	
Unsatisfactory	57	45.2	21	50.0	36	42.9	

CCPG: chlorhexidine, cetylpyridinium chloride and propolis extract group; NS: Not significant; CPOHI: critical patient oral hygiene index. *Some patients had more than one alteration.

Other frequent intraoral findings included biofilm accumulation and dental calculus (41.3%), residual root (21.4%), and caries (19.0%), with no differences in frequency between the groups. Most patients were partially edentulous (67.5%).

Table 3 also shows the frequency of patients classified according to the CPOHI, stratified by study group. Oral hygiene was performed by the nursing team in 92.1% of the patients during hospitalization, and oral hygiene quality was satisfactory in 54.8% and unsatisfactory in 45.2% of the patients, with no statistical differences between the groups. Median CPOHI values were 1.5 (minimum: 0; maximum: 5; interquartile range (IQR): 3) for the RefG and 1.0 (minimum: 0; maximum: 5; IQR: 2, p = 0.075) for the CCPG.

Evaluation of the total sample revealed that the RefG was associated with worse prognostic scores, as indicated by higher median scores SAPS 3 (median RefG 66.5 x CCPG 49.0; p = 0.006) and SOFA Dv (median Ref 5.5 x CCPG 2.0; p = 0.002). When satisfactory levels of hygiene were observed, worse prognostic scoreswere observed for SOFA Dv in the RefG compared with the CCPG (median RefG 4.0 x CCPG 1.0, p=0.001). (Table 4)

**Table 4:** Median prognostic scores stratified by level of oral hygiene (CPOHI) and study group in the 126 studied patients.

Oral hygiene level (CPOHI)	Reference group*		CCPG**		p- value***
	n = 42		n = 84		
	median (min-max)	interquartile range	median (min-max)	interquartile range	
Satisfactory					
SAPS 3	57.0 (32-98)	33.0	50.5 (31-89)	19.0	0.142
SOFA D1	4.0 (0-12)	6.0	2.5 (0-13)	5.0	0.245
SOFA Dv	4.0 (0-13)	8.0	1.0 (0-13)	3.0	0.001
Unsatisfactory					
SAPS 3	71.0 (23-101)	35.0	47.0 (26-94)	31.0	0.030
SOFA D1	8.0 (0-18)	10.0	7.0 (0-15)	8.0	0.171
SOFA Dv	6.0 (0-18)	9.0	6.0 (0-14)	10.0	0.411
Total					
SAPS 3	66.5 (23-101)	32.0	49.0 (26-94)	23.0	0.006
SOFA D1	5.0 (0-18)	7.0	3.0 (0-15)	7.0	0.060
SOFA Dv	5.5 (0-18)	8.0	2.0 (0-14)	6.0	0.002

CCPG: chlorhexidine, cetylpyridinium chloride, and propolis extract group; CPOHI: Critical patient oral hygiene index; SAPS 3: simplified acute physiology score; SOFA D1: sequential organ failure assessment – on the day of admission; SOFA Dv: sequential organ failure assessment – on the day of examination; min-max: minimum-maximum. *Reference group, comparison between satisfactory and unsatisfactory hygiene: SAPS 3, SOFA D1, and SOFA Dv were not significant; **Chlorhexidine group, comparison between satisfactory and unsatisfactory hygiene: SOFA Dv p = 0.001; SAPS 3 and SOFA D1 were not significant. ***Comparison between groups.

**Figure 2:**
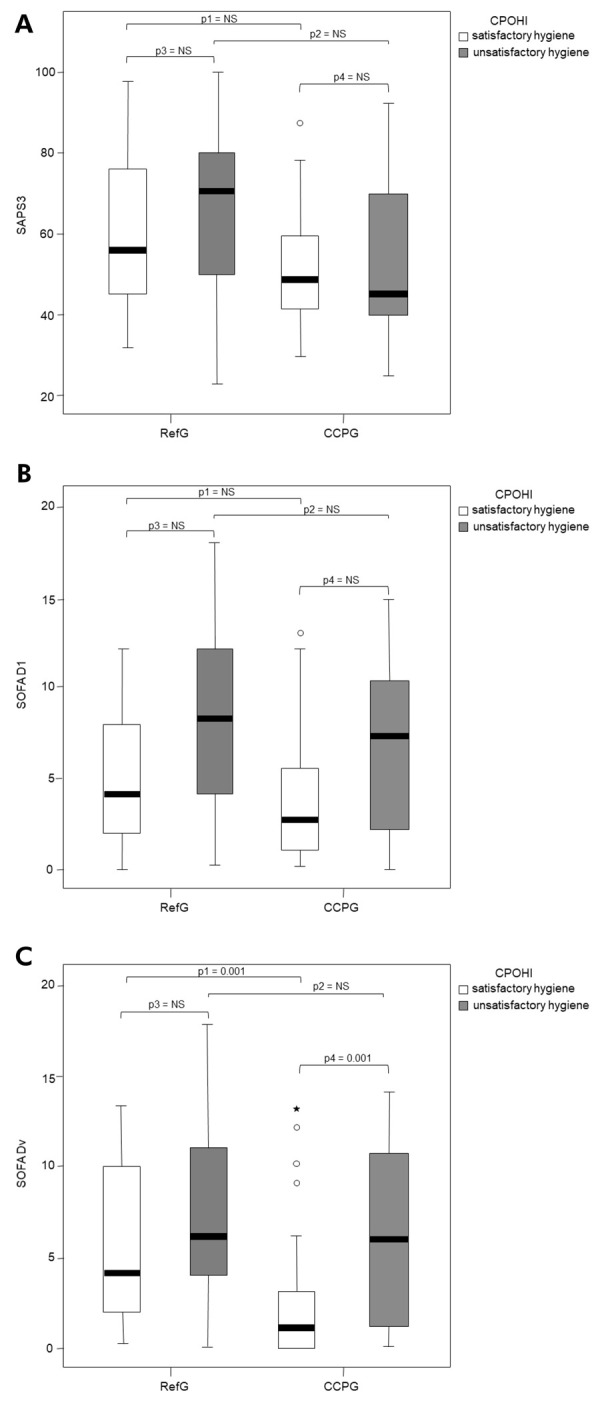
Prognostic scores of patients in the reference group (RefG) and the chlorhexidine, cetylpyridinium chloride, and propolis extract group (CCPG) according to oral hygiene levels assessed by the CPOHI: (a) SAPS3 values, (b) SOFA D1 values, and (c) SOFA Dv values. CPOHI: critical patient oral hygiene index; SAPS3: simplified acute physiology score; SOFA D1: sequential organ failure assessment – on the day of admission; SOFA Dv: sequential organ failure assessment – on the day of examination. RefG: reference group; ChlG: chlorhexidine-based oral rinse group; p: p-value; NS: not significant.

Figure 2 shows boxplot graphs with between-group and within-group comparisons, based on oral hygiene status. When patients in the CCPG were evaluated separately and stratified by oral hygiene status, worse prognostic SOFA Dv scores were observed among those with unsatisfactory oral hygiene (satisfactory 1.0 x unsatisfactory 6.0; p = 0.001). When the same analysis was applied to the RefG, no significant differences in prognostic scores were observed among patients with different levels of oral hygiene.

In the constructed GLM, exposure to the antimicrobial rinse was associated with a protective effect on SOFA Dv scores, whereas poor oral hygiene exerted a negative influence (data not shown). The influence of age, intubation, length of ICU stay, underlying diseases, oral hygiene status, and exposure to the antimicrobial rinse on each prognostic score was evaluated using the constructed GLM. When all variables were included in the model, intubation was the only factor that significantly and negatively influenced all prognostic scores. Oral nutrition exerted a protective effect across all evaluated scoring systems (Tables 5 [Table t6] through 7).

**Table 5:** Direct effects of the full general linear model on the relationships between SAPS 3 and comorbidities, exposure to antimicrobial rinse, oral hygiene, intubation, and nutritional status.

Dependent variable: SAPS3				
Parameter	β bootstrapped coefficient	p-value	99%CI	
			Lower bound	Upper bound
CCPG	1.265	0.626	-5.523	8.053
Unsatisfactory hygiene	-1.633	0.506	-8.039	4.772
Intubation	-11.452	0.001	-20.273	-2.631
Oral nutrition	13.307	0.000	5.396	21.218
Comorbidities				
Heart diseases	-5.825	0.085	-14.600	2.949
Malignant neoplasms	-16.240	0.000	-23.310	-9.170
Diabetes	-.914	0.727	-7.752	5.923
Kidney diseases	-9.377	0.003	-17.549	-1.206
Respiratory diseases	-1.932	0.544	-10.238	6.375

CCPG: group exposed to oral rinse containing chlorhexidine, cetylpyridinium chloride, and propolis extract.

**Table 6 t6:** Direct effects of the full general linear model on the relationships between SOFA D1 and comorbidities, exposure to antimicrobial rinse, oral hygiene, intubation, and nutritional status.

Dependent variable: SOFA D1				
Parameter	β bootstrapped coefficient	p-value	99%CI	
			Lower bound	Upper bound
CCPG	-0.657	0.362	-2.538	1.223
Unsatisfactory hygiene	-1.271	0.063	-3.046	0.503
Intubation	-2.912	0.002	-5.355	-0.468
Oral nutrition	4.069	0.000	1.878	6.260
Comorbidities				
Heart diseases	-1.038	0.266	-3.469	1.392
Malignant neoplasms	-1.551	0.040	-3.510	0.407
Diabetes	.615	0.397	-1.279	2.509
Kidney diseases	-1.595	0.067	-3.859	0.668
Respiratory diseases	-.023	0.979	-2.324	2.278

CCPG: group exposed to oral rinse cointaining chlorhexidine, cetylpyridinium chloride, and propolis extract.

**Table 7:** Direct effects of the full general linear model on the relationships between SOFA Dv and comorbidities, exposure to antimicrobial rinse, oral hygiene, intubation, and nutritional status.

Parameter	β bootstrapped coefficient	p-value	99%CI	
			Lower bound	Upper bound
CCPG	0.064	0.915	-1.507	1.635
Unsatisfactory hygiene	-0.520	0.360	-2.002	.962
Intubation	-5.074	0.000	-7.116	-3.033
Oral nutrition	4.063	0.000	2.232	5.893
Comorbidities				
Heart diseases	-0.057	0.942	-2.087	1.974
Malignant neoplasms	0.349	0.578	-1.287	1.985
Diabetes	0.133	0.827	-1.450	1.715
Kidney diseases	-1.508	0.039	-3.399	0.383
Respiratory diseases	-0.538	0.465	-2.460	1.384

CCPG: group exposed to oral rinse cointaining chlorhexidine, cetylpyridinium chloride, and propolis extract.

## Discussion

The present study comparatively evaluated critically ill patients who received oral hygiene care either with or without a rinse containing 0.12% CCP. The combined use of these antimicrobial agents in oral hygiene formulations has been employed to enhance antiseptic efficacy and reduce dental plaque formation, often with the aim of achieving synergistic effects among the components. ^27,28^ No differences were observed between the groups in the percentage of patients with different levels of oral hygiene, as assessed by the CPOHI. Better SOFA Dv prognostic scores were observed in the group exposed to the antimicrobial oral rinse. These findings suggest that, despite the absence of differences in the percentage of patients with different oral hygiene levels between groups, the use of the antimicrobial oral rinse was associated with better prognostic scores.

The present study corroborates the findings of previous studies that have identified oral hygiene as an important indicator of health and well-being in hospitalized patients.^3,37,38^ Among those patients exposed to the antimicrobial rinse, unsatisfactory oral hygiene was associated with worse SOFA Dv scores. These findings highlight the importance of appropriate oral hygiene in critically ill patients, as poor hygiene may be associated with worse prognostic scores.

In the constructed GLM, exposure to the antimicrobial rinse was associated with a protective effect on SOFA Dv scores, whereas poor oral hygiene showed a negative effect. Nevertheless, in the fully adjusted model including all relevant variables, intubation was the only factor that negatively influenced all analyzed scores, while oral nutrition showed a positive effect across all scoring systems. Therefore, intubation appears to be the main parameter associated with worse prognostic scores. The protective effect of oral nutrition may be attributed to the absence of intubation and, consequently, better prognostic performance.

Of note, the SOFA scoring system is more representative, as it provides measurements at two distinct time points: D1 and Dv. Therefore, any expected influence of the antimicrobial oral rinse and oral hygiene status on prognostic scores would be more appropriately reflected by SOFA Dv, considering that SAPS 3 is assessed only at D1, when patients have not yet been exposed to the antimicrobial solution.

Although SAPS 3 is calculated using baseline data, such as patient characteristics, indication for ICU admission, and physiologic derangement at ICU admission, it reflects the patient’s prognosis in the ICU and is considered a predictor of hospital mortality at the time of ICU admission.^29^ The significant difference in SAPS 3 scores between groups may be regarded as a limitation of the study; however, this difference was not observed when the groups were stratified by oral hygiene levels.^29,30^ Factors such as underlying diseases, length of ICU stay, intubation, level of consciousness, and laboratory test results may also influence these prognostic scores.^9,10^In fact, higher frequencies of respiratory and renal complications were recorded among individuals in the RefG. In the fully adjusted model, malignant neoplasms and renal diseases were associated with lower SAPS 3 scores, which may explain the higher SAPS 3 scores in the RefG.

Among hygiene-related intraoral alterations, gingival inflammation and spontaneous bleeding were significantly more prevalent in the RefG. In the present study, comprehensive periodontal examinations were not performed; however, the observed gingival inflammation is one of the clinical signs of gingivitis, a mild periodontal disease. Periodontal disease has been linked to worse systemic conditions,^31^ and may be aggravated in intensive care patients.^32^ These findings highlight the importance of incorporating chlorhexidine into oral hygiene protocols for critically ill patients, as supported by the literature.^13,14^

The absence of significant differences between groups regarding oral findings such as calculus, caries, fracture, residual root, and tooth mobility, may be explained by the inclusion of patients hospitalized for at least 5 days, given that such changes develop over longer periods and depend on patients’ oral health status at baseline.

The use and effectiveness of chlorhexidine in preventing oropharyngeal colonization and reducing respiratory complications in ICU patients have been widely investigated.^8^ Several studies have demonstrated an association between chlorhexidine use and a reduction in ventilator-associated pneumonia,^2,8^ as well as a potential relationship between oral hygiene and the deterioration of systemic conditions in ICU patients.^13,14^ In the present study, all included patients stayed at least five days in the ICU, indicating that patients in the CCPG were exposed to antimicrobial oral rinse (CCP) for at least five days. The use of CCP was expected to result in improved oral outcomes and, consequently, better SOFA Dv scores in the CCPG, as actually observed.

Recently, a meta-analysis has suggested a trend toward increased mortality among non-cardiac surgical patients treated with chlorhexidine-based oral rinse.^33^ It has been suggested that aspiration of chlorhexidine by some patients may have led to acute respiratory distress syndrome or an allergic reaction, including anaphylaxis. Accordingly, recent studies have questioned the use of chlorhexidine in oral hygiene protocols for mechanically ventilated patients.^34,35^ Therefore, the identification of patients who may or may not benefit from chlorhexidine use in oral hygiene protocols should be guided by scientific evidence, with the aim of reducing the risk of acquired chlorhexidine resistance or cross-resistance to antibiotics.^36^

While the study population was heterogeneous, this drawback was outweighed by the comparability of demographic variables and the frequency of underlying diseases. Consistent with the inherent limitations of case-control studies, a definitive cause-and-effect relationship cannot be established, with conclusions confined to associations between variables. Multiple systemic factors may influence the general health of hospitalized patients. In addition, patients who are alert and oriented, breathing spontaneously and receiving oral feeding, have better cognitive functions and possibly perform oral hygiene more effectively.

Bedside examination also posed some challenges in the present study, notably in intubated patients, given the presence of an orotracheal tube and limited mouth opening. Although oral hygiene was performed by multiple nursing professionals and adherence to oral hygiene procedures and rinse application was not directly monitored, all professionals attended the same oral hygiene training course and watched the same instructional video developed by the dental research team. Moreover, no significant differences were observed between groups regarding oral hygiene levels, as classified by the CPOHI, suggesting similar performance of the oral hygiene protocol by the nursing staff.

Recent discussions and questions about the use of antimicrobial rinses contribute to scientific advancements and underscore the need for further randomized clinical trials, with the participation of dentists in the study groups and multidisciplinary teams, for the establishment of safe and scientifically grounded guidelines focused on patient well-being.

## Conclusion

In this inpatient population of an ICU, those exposed to antimicrobial oral rinse exhibited better SOFA DV scores than the patients in the reference group. Among the patients exposed to the oral rinse, those with better oral hygiene status showed better SOFA Dv prognostic scores. In the multivariate analysis, intubation was the only factor with a negative effect on all prognostic scores. These findings underscore the importance of oral hygiene protocols that include antimicrobial rinse for critically ill patients, highlighting the role of dentists as integral members of the ICU multidisciplinary care team.

## Data Availability

The authors declare that all data generated or analyzed during this study are included in this published article.
